# Faculty bridging individual and organizational resilience: results of a qualitative analysis

**DOI:** 10.1080/10872981.2023.2184744

**Published:** 2023-03-02

**Authors:** Meera Deva, Gary L. Beck Dallaghan, Neva Howard, Brenda J.B. Roman

**Affiliations:** a School of Medicine, University of North Carolina, Chapel Hill, USA; b, University of Texas at Tyler School of Medicine, Tyler, TX, USA; c School of Medicine, Wright State University Boonshoft, USA

**Keywords:** Resilience model, medical students, faculty, medical schools, academic affairs

## Abstract

**Background:**

Medical student burnout and anxiety has received growing attention in the past decade. The culture of competition and assessment has resulted in increasing stress levels amongst medical students, causing a decline in their academic performance and overall mental health. The objective of this qualitative analysis was to characterize recommendations from educational experts to aid students’ academic progress.

**Methods:**

At an international meeting in 2019, worksheets were completed by medical educators during a panel discussion. Participants responded to four scenarios representing common challenges medical students face in school (eg. Postponing Step 1, failing clerkships, etc.). For each case, participants addressed what students, faculty and medical schools could do to mitigate the challenge. Inductive thematic analysis was conducted by two authors followed by deductive categorization using an individual-organizational resilience model.

**Results:**

Across the four cases, common suggestions made for students, faculty and medical schools were aligned to a resilience model representing the complex interplay between individuals and organizations and the impact on student wellbeing.

**Discussion:**

Using suggestions from medical educators from across the US, we were able to identify recommendations for students, faculty, and medical schools to help students succeed in medical school. By applying a model of resilience, faculty serve as a critical bridge to connect students to the medical school administration. Our findings also support a pass/fail curriculum to ease the competition and burden students place on themselves.

## Introduction

Medical student life is challenging – juggling intense course schedules, long hours studying, involvement in extra-curricular activities, and the burdens of daily life. Other stressors, such as achievement goals [1] and the culture of assessments [2], impact medical student performance. It is not surprising that studies have found increasing levels of anxiety and stress in college populations [3] as well as medical students [4]. These stressors may ultimately lead to burnout and other mental health crises [5], which has motivated medical schools to identify strategies to help students.

One study highlighted factors such as extensive course load, lack of exercise and long duration of exams as contributing factors to exam anxiety [6]. Similarly, a study looking at the relationship between medical student emotions and burnout found positive associations between academic performance with pride and hope, but negative associations with anxiety and shame. This leads us to believe that if we can better understand contributors to medical student achievement emotions [7], we can potentially improve confidence and academic performance [8]. Achievement emotions include enjoyment, hope, pride, anger, shame boredom, anxiety and hopelessness [9]. If contributors to stress include standardized exams, or other methods of critical assessment, then it is worthwhile to explore changes to these systems if it contributes to an improvement in the mental health of students [10].

The stressors impact not only the individual learner but also the organization [5]. When students have academic struggles, organizations are obligated to respond. Providing remedial opportunities for students comes with a significant administrative burden, however [11]. In addition to identifying strategies for individuals, it is also important to consider organizational strategies that could help contribute to a sense of resiliency.

There has been an increased interest in resilience because of its emphasis on promoting coping, adapting, and thriving from adverse events [12]. Definitions of resilience point out that it is not merely the individual that must address the adversity. There is a complex interplay between the individual, environment, and socio-cultural factors impacting resiliency [5]. Adapting and coping to adversity has tended to fall on individuals to address [13,14]. However, as noted by Venegas and colleagues [12], resilience relies on the synergy of the individual and the organization.

Peer review literature over the past 5 years has seen an increase in the numbers of publications related to wellness initiatives to address burnout amongst health care professionals [5]. The Alliance for Clinical Education (ACE) conducted a workshop at the 2019 Association of American Medical Colleges (AAMC) Learn Serve Lead conference to explore potential solutions to this problem. Our aim was to obtain participant input on steps medical students, faculty, and organizations can take to mitigate stressors.

## Methods

ACE presented a panel discussion entitled ‘Testing Drives Curriculum but Does it Drive Anxiety?’ At the 2019 AAMC Learn Serve Lead meeting to approximately 100 medical educators. ACE is an organization comprised of representatives from eight clinical medical student education organizations (emergency medicine, family medicine, internal medicine, neurology, OB/GYN, pediatrics, psychiatry and surgery). These representatives are from medical schools across the US and are clerkship directors or assistant/associate deans. The objectives of the panel discussion were to: 1) Identify the main sources of anxiety students have regarding assessments; 2) List the attributes of millennial and Gen Z students that might further contribute to their anxiety and 3) Identify ways to mitigate this anxiety in the culture of assessment.

Participants attending the panel discussion come from medical schools or healthcare systems primarily in the US or Canada. The AAMC Learn Serve Lead meeting attracts medical students, residents in training, faculty, deans, education administrators, and other leaders in medical education. Participants are involved with undergraduate, graduate and continuing medical education. Any of these individuals may have been present for the panel discussion. Due to the transient nature of these sessions with people coming and leaving, we chose to not attempt to collect demographic data as we would not have been able to capture accurate data.

To address the various stressors, we had participants working in small groups at the round tables in the room. Participants were provided worksheets detailing four different case scenarios developed by the leaders of the session. The cases represent common issues the session leaders have identified frequently at their institutions. The case scenarios are summarized in Appendix A. Participants wrote down suggestions about how students could respond to the scenario, how faculty could respond, and how the organization could respond during the session. Participants did share their ideas during large group discussions. The worksheets were collected at the end of the panel discussion and did not include any personal or other identifiable information.

### Data analysis

We used inductive thematic analysis to identify patterns within and across the collected data to construct meaning [15]. We combined comments from the completed worksheets into a spreadsheet for coding and analysis. Two authors (MD, GLBD) independently coded all transcribed comments inductively. We met to resolve coding discrepancies and our interpretation of the interconnectedness of codes. After further discussion, the authors felt the individual and organizational resilience model proposed by Vercio and colleagues [5] could best organize and describe our findings. Therefore, we deductively categorized our themes based on constructs identified in the individual and organizational resilience model.

**Figure 1.**
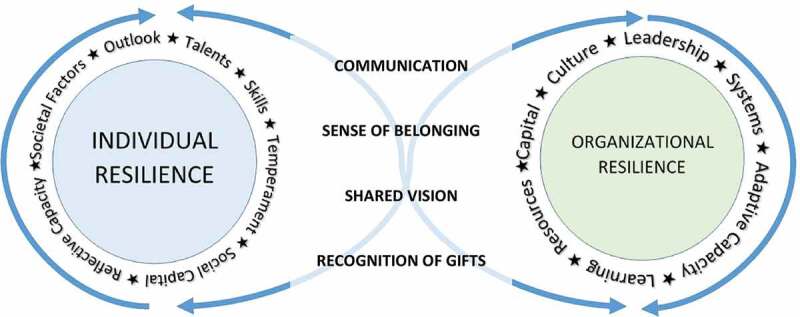
Individual and organizational resilience model (Adapted from Vercio et al.).

The individual and organizational resilience model describes the interplay between the two as interdependent (Figure 1). Stressors in the model impact both individuals and organizations. Factors associated with individuals are categorized as internal (e.g., temperament, outlook, talents, skills, reflective capacity), social capital, and societal factors. Dimensions of organizational resilience included culture, social networks, learning, leadership, resources, adaptive capacity, systems and capital. Dimensions bridging individual and organization are described as communication, sense of belonging, shared vision, and recognition of gifts. Using this model, we were able to construct greater meaning from our results by deductively aligning codes to elements of this model.

This study was approved by the Institutional Review Board at the University of North Carolina as exempt.

## Results

The space reserved for this session was designed to accommodate 150 participants. An initial count at the beginning of the session totaled roughly 85–90 participants, but people come and go from these sessions so a final count could not be calculated. A total of 33 worksheets were returned. Most tables had 3 to 4 participants working together, but some participants chose to work alone.

The inductive coding resulted in 13 themes that were categorized by student action, faculty action and institutional action (Table 1).

**Table 1.** Themes associated with individual and organizational resilience model.

Inductive Themes	Resilience Model Factors
Students:	
Seek help, Take Responsibility	Social Capital, Outlook
Respond to help	Temperament, Reflective Capacity
Master study strategies	Skills, Reflective Capacity
Perspective shift	Outlook
Faculty:	
Building trust	Sense of Belonging
Improve communication	Communication
Normalize academic struggles	Shared Vision
Create realistic expectations	Recognition of Gifts
Institution:	
Change learning environment	Adaptive capacity
Improve feedback	Resources
Formalize academic excellence	Capital, Culture
Create community	Leadership
Set expectations	Systems

A common theme for student action was for students to seek help from faculty, advisors, or peers early and follow-up with the support given. Some participants suggested that asking for help and responding to help given is a professional act.

- ‘should have sought early interventional support about below average scoring. He should have engaged with offered support’ -Participant 33
- ‘Never stop reaching out – no way to assess the reasons that way – and an aspect of professionalism.’ -Participant 13

Another theme for students was to master their study strategies and practice methods early. Advice on where to seek help was given by a few, such as the Office of Academic Excellence, learning specialists, upperclassmen, and peer mentors. A final theme for students involved a perspective shift. The participants recommended students learn methods of anxiety mitigation and understanding that asking for help is not a weakness.

- ‘See [promotions] committee as wanting to help not punish’ -Participant 7
- ‘Students should take the long view on how to do well in medical school with life balance (eg, time management, envisioning the future) vs what they can do at the last minute’ -Participant 27
- ‘Get therapy and outside help for anxiety/imposter syndrome’ -Participant 22

For faculty and advisors, building trust and improving communication with students was a theme wherein suggestions to build trust included normalizing academic struggles that medical students face. By normalizing the struggles, students may feel comfortable asking for help and establishing a close relationship with their advisors [16].

- ‘Help students transition through empowerment’ -Participant 10
- ‘More trust between coaches and students’ -Participant 12

Additionally, creating realistic expectations about what to expect in medical school and encouraging a growth mindset were options for advisors to implement.

- ‘Change mindset of students (delaying is OK)’ -Participant 2
- ‘Create realistic expectation about USMLE exams’ -Participant 12

The medical school theme was summarized as changing the learning environment. This included improving feedback mechanisms for students to submit, creating a safe learning environment, formalizing academic excellence programs, and creating community through focus groups, tutors, faculty, and wellness programs.

- ‘Build trust and set up appropriate process for communication with students’ -Participant 1
- ‘Learning communities fostering support’ -Participant 13
- ‘Have a better system of student support and a better standard operating procedure’ -Participant 19
- ‘School should offer a pre-matriculation transition curriculum, which may assist acculturation in medical education and anticipate challenges (and develop strategies for addressing them on student’s own) - this can build trust in the school’ -Participant 27
- ‘School could support students in developing a longer view, eg with assigned advisors, periodic mock exams, time management courses, peer mentors’ -Participant 27

Many participants mentioned setting better expectations at the beginning of courses and clerkships. Specifically, creating a formal outline of expectations and assessment plans to share on the first day. Mid-clerkship, participants suggested a ‘check-in’ process for the students to communicate with their advisors about their fear, imposter syndrome, and the learning environment. OSCE’s were also recommended by many participants.

- ‘Provide some space for formative feedback and vulnerability’ -Participant 12
- ‘Be honest about expectations on rotations’ -Participant 22

As noted in the Methods, a deductive approach was taken with the themes to classify them using the individual and organizational resilience model [5]. What we found was that suggestions for student actions fit aspects of individual resilience (Table 1) and institutional actions fit within organizational resilience. The themes constructed for faculty action bridged individual and organization.

## Discussion

Themes constructed from participant input suggested students need to seek assistance early, ‘creating social capital and improving outlook’ in the words of Vercio and colleagues [5]. In order for students to feel safe and supported, medical schools need to establish a learning environment that is psychologically safe [17,18]. Faculty need to acknowledge that asking for help may be very hard for students that are used to being the high performers in undergraduate education, so medical schools need to make sure that there are numerous ways to ask for help, perhaps including the training of student ‘ambassadors’ to share their experiences with the resources available. The perspective shift of developing a growth mindset and that we all learn more from our struggles than our successes needs to be encouraged early in medical school [19]. This also needs to be modeled by the teaching faculty! This role modeling could be interpreted as a bridging shared vision (Figure 1).

With the United States Medical Licensing Examination (USMLE) Step 1 and other nationally standardized examinations now being pass-fail, medical schools have an opportunity to adopt curricula that fosters academic excellence in a nurturing environment. The learning environment should encourage cooperative learning, not competition amongst the students. Studies have shown that pass-fail frameworks contribute to cooperate learning environments as well as mastery learning [20–22]. Academic support personnel, including physician educators, need to be equipped with how to best support students, and work with mental health counselors when indicated. These centers should hire enough staff so that all students can be adequately served – as sometimes even the best medical students need additional support [11].

The individual-organizational resilience model involves a synergistic relationship [5]. Therefore, it is not enough to suggest actions for faculty and the medical school without addressing what students need to do. As our participants indicated, medical students need to be proactive, seeking help and being open to feedback [19]. In order to develop a growth mindset, individual actions identified by our thematic analysis, such as seeking help, responding to help, and perspective shifts, are essential.

Based on responses from participants, faculty and advisors play a major role in student self-esteem. Per the themes identified in the medical school category, faculty and advisors are responsible for being student advocates and acting as the bridge between the medical school and students. By improving the means of communication that students have to voice their concerns, and by normalizing academic struggles, faculty can create a reliable community for students to lean on in times of need.

Additionally, in order to further promote medical student well-being, there are necessary changes that need to be made on an individual (advisor) level as well as institutional level. By acting on the themes represented in the resilience model, medical schools can facilitate student growth. They will need to not only make curriculum-based changes such as pass/fail, but also improve the training of faculty/advisors to adopt counseling styles that recognize the strengths of students and help them create a sense of belonging in the medical school.

A limitation of this study is that the data was collected at a conference that presents logistical challenges to obtain accurate demographic data about participants. Although participants at the conference have an interests in medical education in general, there may be selection bias for our session as participants chose to attend amidst the numerous other sessions being offered at the same time. However, we feel that many of the findings we constructed through our thematic analysis are reflected in the literature, and are unique in that we have deliberately applied a model of individual and organizational resilience to the results. Since we were uncertain if medical students were present, future work should deliberately include their input. Future endeavors should also consider if the faculty bridge between individual and organization is unique depending on if faculty are pre-clinical or clinical educators.

## Conclusions

We believe that this study offers important insights into the framing of medical education from multiple perspectives. After characterizing recommendations from educational experts, it is evident that the role of students and organizations in academic success is interdependent. Additionally, faculty play an important role in strengthening the connection between institutions and students, while also maintaining their advisory role. With the ultimate goal of increasing academic outcomes and student well-being, medical school efforts need to be focused on strengthening faculty and student relationships, changing the curriculum in pre-clinical and clinical years to accommodate burnout, and facilitating an encouraging learning environment by increasing student avenues of communication with medical schools. Our qualitative study emphasizes how students, institutions, and faculty all have an interdependent network that can contribute to the larger, positive learning environment, and it is important for future research to explore this interplay further, potentially after a few more years of a pass-fail USMLE Step 1.

## Data Availability

The data that support the findings of this study are available from the corresponding author, GLBD, upon reasonable request.

## Statements and declarations

Dr. Beck Dallaghan is the executive director for the Alliance for Clinical Education; however, he does not receive financial compensation for this role.

## APPENDIX

**Table:**

CASE A: First year obstacles	Martin Jones grew up in a small rural town in western NC. In high school, he was a straight A student and played multiple varsity sports. He decided to defer college as he felt that he needed to earn money to support his family, and so began working as a nursing assistant at the regional hospital close to his hometown. Two years after graduation, he started community college and then transferred to Appalachian State University, a public undergraduate institution with about 5,000 students per year. He again excelled academically and became the student body president. His college advisor suggested medicine as a career option which surprised and, eventually, intrigued Martin. He entered medical school the following year and became the MS1 representative to the curriculum committee. He scored well on his first exam, but then scored well below average on the following few exams. He was asked to appear in front of the Progress Committee. The committee referred him for academic support in the Office of Academic Excellence, but he did not follow through. He stopped attending curriculum committee meetings. His advisor reached out to him but did not hear back. He failed his Medical Science course and is now again in front of the Progress Committee.
CASE B: Step 1 Delay	At Universal Medical School, Step 1 is taken at the end of the Foundations Phase of the curriculum, prior to starting core clinical clerkships in April. The students finish with their core curriculum in mid-February and have up to 7 weeks of dedicated Step 1 study time. For the last ten years, about 10% of the class has delayed taking Step 1 due to not likely being able to receive a passing score based on their Comprehensive Basic Science Self-Assessment ([CBSSA], taken 2 weeks into dedicated study time) and their overall performance in the curriculum. However, in 2019, 28% of the class delayed. Based on academic performance in the curriculum, school administration felt that only about 15% should have delayed. In meetings with students, they cited increasing anxiety and desire to ‘get a higher score,’ so purposely did not do well on the CBSSA. They also cited ‘not feeling the school properly prepared them since the curriculum was new.’ The process of getting approvals for delays included submitting a completed CBSSA score. Some scores were noted to be suspiciously low, but individuals responsible for approval felt in a bind – if they denied a request and the student ultimately failed, they felt they would be to blame for the failure. Faculty also noted increasing class anxiety about the exam, and despite trying to calm fears, felt it was challenging to manage the ‘anxiety ridden Step 1 culture’. The administration, including clerkship directors, do not want another year of nearly 30% of the class delaying, so are wanting suggestions to prevent this from happening again.
CASE C: Third year Rotations	Samantha Conrad is a third-year medical student who has just completed her first clinical rotation. During her pre-clinical years at Universal Medical School, she performed above average on her course exams and received high marks in her clinical skills course. After taking the USMLE Step 1 exam, she was thrilled to finally head into her clinical rotations and apply the knowledge she had learned during her pre-clinical years. However, during her first clinical rotation in Internal Medicine, she found it difficult to learn from and enjoy her time on the wards due to her anxieties about being constantly evaluated by others. She avoided asking questions for fear that they would be viewed as ‘stupid’ and avoided answering questions for fear of being wrong. When presented with opportunities to lead a patient interview, she worried about saying or asking the wrong thing and found herself barely able to complete the interview. At the end of her rotation, she did not seek in-person feedback from her attendings and residents with whom she worked because of her fears about what they had to say about her. In her formal feedback, her attendings stated that overall, she performed ‘below the level of expectation for a third-year medical student.’ Samantha is now about to start her obstetrics and gynecology rotation and she is looking forward to this rotation as she has a strong interest in this field. However, she has become increasingly anxious about her upcoming rotation and is unsure how to perform better and make the rotation a better experience.
CASE D: Step 2 Clinical Skills	Having completed required clerkships, Jordan Cohen has been enjoying taking electives related to his chosen career during his final year of medical school. He did well on Step 1 and Step 2 Clinical Knowledge and only has Step 2 Clinical Skills left to take. Thinking that this exam is similar in nature to some of the OSCEs he has taken throughout medical school, he has not been spending a lot of time studying for the exam. However, a few weeks before his exam scheduled for January, he begins hearing from others about how hard the test is and even, that some students are failing it. He is growing more anxious thinking that he is not prepared for the test, but no other dates are available for him to switch to. He begins to wonder: What happens if I fail? Will the scores be out prior to the Match and impact my chances of getting the residency I want? Several days before the test, he is frantically studying the materials from the USMLE website. He goes into the examination feeling as though he is not prepared at all and fears he might fail.

## References

[1] Artino AR, La Rochelle JS, Durning SJ. Second-year medical students’ motivational beliefs, emotions, and achievement. Med Educ. 2010;44(12):1203–7. 21091760 10.1111/j.1365-2923.2010.03712.x

[2] Khalil MK, Williams SE, Hawkins HG. Learning and study strategies correlate with medical students’ performance in anatomical sciences. Anat Sci Educ. 2018 Accessed October 25, 2019;11(3):236–242. 28940743 10.1002/ase.1742

[3] Beiter R, Nash R, McCrady M, et al. The prevalence and correlates of depression, anxiety, and stress in a sample of college students. J Affect Disord. 2015 Mar 1;173:90–96. Epub 2014 Nov 8. PMID: 25462401. 25462401 10.1016/j.jad.2014.10.054

[4] Ishak W, Nikravesh R, Lederer S, et al. Burnout in medical students: a systematic review. Clin Teach. 2013 Aug;10(4):242–245. PMID: 23834570. 23834570 10.1111/tct.12014

[5] Vercio C, Loo LK, Green M, et al. Shifting focus from burnout and wellness toward individual and organizational resilience. Teach Learn Med. 2021;33(5):568–576. Epub 2021 Feb 15. PMID: 33588654. 33588654 10.1080/10401334.2021.1879651

[6] Hashmat S, Hashmat M, Amanullah F, et al. Factors causing exam anxiety in medical students. J Pak Med Assoc. 2008 Apr;58(4):167–170. PMID: 18655422. 18655422

[7] Beck G Investigation of the relationship between achievement emotions and academic performance in medical students. 2011. https://pqdtopen.proquest.com/doc/894260388.html?FMT=ABS

[8] Burr J, Beck Dallaghan GL. The relationship of emotions and burnout to medical students’ academic performance. Teach Learn Med. 2019 Oct-Dec;31(5):479–486. Epub 2019 May 22. PMID: 31116577. 31116577 10.1080/10401334.2019.1613237

[9] Pekrun R, Goetz T, Frenzel AC, et al. Measuring emotions in students’ learning and performance: the Achievement Emotions Questionnaire (AEQ). Contemp Educ Psychol. 2011;36(1):36–48.

[10] Zeller A, Handschin D, Gyr N, et al. Blood pressure and heart rate of students undergoing a medical licensing examination. Blood Press. 2004;13(1):20–24. 15083636 10.1080/08037050310025645

[11] Kalet A, Chou CL, Ellaway RH. To fail is human: remediating remediation in medical education. Perspect Med Educ. 2017 Dec;6(6):418–424. 29071550 10.1007/s40037-017-0385-6 PMC5732108

[12] Venegas CL, Nkangu MN, Duffy MC, et al. Interventions To improve resilience in physicians who have completed training: a systematic review. PLoS ONE. 2019 Jan 17;14(1):e0210512. 30653550 10.1371/journal.pone.0210512 PMC6336384

[13] de Lint W, Chazal N. Resilience and criminal justice: unsafe at low altitude. Crit Crim. 2013;21:157–176.

[14] Goroll AH. Addressing burnout-focus on systems, not resilience. JAMA Netw Open. 2020 Jul 1;3(7):e209514. 32614420 10.1001/jamanetworkopen.2020.9514

[15] Braun V, Clarke V, Camic PM, et al. Thematic analysis. In: Cooper H Rindskopf D, editors. APA handbook of research methods in psychology, Vol. 2. Research designs: quantitative, qualitative, neuropsychological, and biological. Washington, DC: American Psychological Association; 2012. pp. 57–71.

[16] Zhang Q, Fiorella L. An integrated model of learning from errors. Educ Psychol. 2022;58:18–34. ePub ahead of print.

[17] Tsuei SH, Lee D, Ho C, et al. Exploring the construct of psychological safety in medical education. Acad Med. 2019 Nov;94(11S Association of American Medical Colleges Learn Serve Lead: Proceedings of the 58th Annual Research in Medical Education Sessions):S28–35. PMID: 31365407. 31365407 10.1097/ACM.0000000000002897

[18] McClintock AH, Kim S, Chung EK. Bridging the gap between educator and learner: the role of psychological safety in medical education. Pediatrics. 2022 Jan 1;149(1):e2021055028. PMID: 34972228. 34972228 10.1542/peds.2021-055028

[19] Dweck CS. Mindset: the new psychology of success. New York: Ballantine Books; 2016.

[20] Reed DA, Shanafelt TD, Satele DW, et al. Relationship of pass/fail grading and curriculum structure with well-being among preclinical medical students: a multi-institutional study. Acad Med. 2011 Nov;86(11):1367–1373. PMID: 21952063. 21952063 10.1097/ACM.0b013e3182305d81

[21] Seligman L, Abdullahi A, Teherani A, et al. From grading to assessment for learning: a qualitative study of student perceptions surrounding elimination of core clerkship grades and enhanced formative feedback. Teach Learn Med. 2021 Jun-Jul;33(3):314–325. Epub 2020 Nov 24. PMID: 33228392. 33228392 10.1080/10401334.2020.1847654

[22] Cutrer WB, Miller B, Pusic MV, et al. Fostering The development of master adaptive learners: a conceptual model to guide skill acquisition in medical education. Acad Med. 2017;92(1):70–75. 27532867 10.1097/ACM.0000000000001323

